# Sociodemographic features associated with the MoCA, SPPB, and GDS scores in a community-dwelling elderly population

**DOI:** 10.1186/s12877-023-04162-z

**Published:** 2023-09-13

**Authors:** Peng Zhang, Nazhakaiti Abudukelimu, Ayijiamal Sali, Jin-Xia Chen, Min Li, Yan-Yan Mao, Yi Zhu, Qian-Xi Zhu

**Affiliations:** 1 School of Management, Hainan Medical University, Haikou, Hainan 571199 China; 2 Shanghai-MOST Key Laboratory of Health and Disease Genomics, NHC Key Lab of Reproduction Regulation, Shanghai Institute for Biomedical and Pharmaceutical Technologies, Shanghai, 200237 China; 3 Department of Medicine, Kashgar Vocational and Technical College, Kashgar, Xinjiang Uygur Autonomous Region 844099 China; 4 Emergency Department, Affiliated Hospital of Jiangsu University, Zhenjiang, Jiangsu 212000 China; 5 School of Clinical Medicine, Shanghai University of Medicine & Health Sciences, Shanghai, 201318 China

**Keywords:** Montreal Cognitive Assessment, Short physical performance battery, Geriatric Depression Scale, Elderly people, Sociodemographic features

## Abstract

**Background:**

An accurate evaluation of cognitive function, physical health, and psychological health is fundamental for assessing health problems in the elderly population, and it is important to identify the necessity of early therapeutic intervention. The objective of this study was to evaluate the states of mental and physical functions and to investigate the relationships between sociodemographic features and these functions in a community-dwelling elderly population.

**Methods:**

This community-based cross-sectional study was conducted in a suburban district of Shanghai, China. A total of 1025 participants aged 60–89 years underwent investigations of demographic and lifestyle features and a multidimensional geriatric evaluation comprising the Montreal Cognitive Assessment (MoCA), Short Physical Performance Battery (SPPB), and Geriatric Depression Scale (GDS).

**Results:**

The results of the multivariate linear regression models demonstrated that the MoCA and SPPB scores decreased with advancing age (all *P* < 0.01). However, the GDS score did not exhibit an age-related decrease (*P* = 0.09). Both sex and living alone influenced the MoCA score (*P* < 0.01 and *P* = 0.04, respectively), SPPB score (*P* < 0.01 and *P* = 0.04, respectively), and GDS score (*P* < 0.01 and *P* < 0.01, respectively). A higher education level was related to better MoCA and SPPB scores (all *P* < 0.01). Furthermore, age and sex had interactive effects on the MoCA score (*P* = 0.03) and SPPB score (*P* < 0.01). The kernel-weighted local polynomial smoothing curves exhibited similar trends.

**Conclusions:**

It is imperative to develop a more sensitive evaluation of physical function, and to encourage various intellectually and emotionally stimulating social activity strategies to promote healthy aging, especially in elderly women and those living alone who have a low education level.

## Background

The rapid increase in the elderly population has caused severe public health issues worldwide. In China, most elderly individuals live at home. An accurate evaluation of cognitive function, physical health, and psychological health is fundamental for assessing health problems in the elderly population, and it is important to identify the necessity of early therapeutic intervention. Mild cognitive impairment (MCI) represents the preclinical, transitional stage between healthy cognitive aging and dementia, and it affects 10–15% of the population older than 65 years [1]. In approximately 50% of individuals with MCI, the condition progresses to dementia within 5 years [2]. The English and Chinese versions of the Montreal Cognitive Assessment (MoCA) have been validated for detecting cognitive impairment [3–5]. The good sensitivity prompted the MoCA to be a useful tool in the MCI research field [6]. Physical function is a critical component of the evaluation of elderly individuals. The Short Physical Performance Battery (SPPB), a physical performance measure, offers better validity and reproducibility than self-reported measures [7]. Depression is a common psychiatric disorder among the elderly. Decreased physical health and social burden caused by depression worsen the quality of life of individuals [8, 9]. The Geriatric Depression Scale (GDS), which exhibits good quality and reliability, has become one of the most widely used screening tools for depression in the elderly population [10, 11].

However, overall assessments of the physical and mental health of community-dwelling elderly individuals in China are scarce. Furthermore, a complete understanding of the sociodemographic factors that contribute to a reduced functional status in the elderly population is lacking. This community-based study was conducted to explore the general health status of the elderly population and possible influencing factors and to provide some practical suggestions for the prevention of the degeneration of cognitive function, physical health, and psychological health.

## Methods

### Study population

This cross-sectional study was conducted in 2019 using a multistage cluster-stratified sampling method. First, 6 towns were randomly selected from 18 towns in a suburban district of Shanghai. Second, three neighborhood committees consisting of more than 100 households were randomly selected from each town. Third, 60 elderly persons aged 60–90 years, with no history of psychotic disorders or limited mobility, were chosen randomly from each committee. Only one person was selected from each household. If the selected person did not meet the inclusion criteria or refused to participate, he or she was replaced by a resident from the same committee. This study was approved by the ethics committee of Shanghai University of Medicine & Health Sciences (2019-SMHC-01-003).

### Assessment of covariates

After obtaining written informed consent from the participants, uniformly trained investigators collected demographic and lifestyle information through structured face-to-face interviews. Age, sex, education level, occupation, living mode, and dietary, sleeping, drinking, and smoking habits were ascertained. Sex, proper diet, sufficient sleep, and living mode were dichotomous variables: male or female, yes or no, yes or no, and living alone or not living alone, respectively. Educational levels included illiteracy, primary school, junior high school, senior high school, and college or higher. Body mass index (BMI) was classified as underweight (< 18.5 kg/m^2^), normal weight (18.5–23.9 kg/m^2^), and overweight (≥ 24 kg/m^2^). However, only 56 individuals were underweight, and they were classified into the normal weight group based on having similar correlations with the covariates.

### Clinical assessments

The participants underwent a multidimensional geriatric evaluation. Cognitive function was assessed using the MoCA. The sum of all item points produced a total MoCA score ranging from 0 to 30 [5]. MCI was identified using education-specific cutoff points for the total MoCA scores. According to the norms of the MoCA for the Chinese population, the cutoff points were ≤ 13 for illiteracy, ≤ 19 for individuals with primary school education, and ≤ 24 for those with middle school or higher education [12]. Physical function was assessed using the SPPB by balance, speed, and chair stand tests. The overall score range from 0 to 12, and less than 10 is classified as lower physical function (LPF) [7]. The GDS includes 30 items, with a total score range of 0 to 30. The participants with a GDS score higher than 10 were categorized as suffering from depression [13, 14]. The Mini Nutritional Assessment (MNA) includes anthropometric, general, dietary, and subjective assessments. The reference values ranged between 0 and 30. Participants with an MNA score of < 24 were defined as having risk of malnutrition (RMN) [15]. The participants were evaluated bone mineral density (BMD), T-score, and Z-score of the left and right femoral necks. Participants with a T-score of <-2 were defined as being at risk of osteoporosis.

### Statistical analysis

The distributions of the MoCA, SPPB, GDS, MNA, and BMD are listed. Analysis of variance (ANOVA) were applied to compare the differences in the means of the five assessments among the different groups of characteristics, including age, sex, diet, sleep, BMI, education, and living alone. Kernel-weighted local polynomial regression smoothing curves with confidence intervals were performed to explore the correlations between age and the five assessment scores, based on the different covariate groups. The sub-health statuses of the five assessments corresponded to MCI, LPF, depression, malnutrition, and osteoporosis, respectively. Chi-square tests were performed to analyze the distribution of the five sub-health statuses among the different subgroups. Based on the results of the univariate analysis and kernel curves, we further fitted multiple linear regression models, including two-way interaction terms if significance existed, to reveal the relationship between the factors and assessments. All statistical analyses were performed using the SAS 9.4 package (SAS Institute, Cary, NC, USA) and Stata 14 (Stata Corporation, College Station, TX, USA), and statistical significance was defined as *P* < 0.05.

## Results

A total of 1025 participants aged 72.0 ± 5.6 years, including 432 men and 593 women, with complete data were included in the statistical analysis. There were 413, 497, and 115 participants in the 60–69, 70–79, and 80–89 years age groups, respectively. The median (interquartile range) MoCA, SPPB, GDS, MNA, and BMD were 19 (14–23), 11 (9–12), 5 (2–8), 26.5 (24.5–27.5) and 42.7 (39.4–47.8), respectively.

ANOVA demonstrated that, except for GDS (*P* = 0.07 and *P* = 0.24), the other four assessments (MoCA, SPPB, MNA, and BMD) were worst in the older age groups (all *P* < 0.01) or in the lower education groups (all *P* < 0.01). Sex (all *P* < 0.05) was an influencing factor on the results of the five assessments, and females exhibited worse results (Table 1).

**Table 1:** Summary of health indices in different sociodemographic groups

	n	Age			MoCA			SPPB			GDS			MNA				BMD		
		Mean	P*		Mean	P*		Mean	P*		Mean	P*		Mean		P*		Mean		P*
Range		60–89			1–30			1–12			0–30			17.5–30				26.9–79.3		
IQR		67–76			14–23			9–12			2–8			24.5–27.5				39.4–47.8		
Medium		71			19			11			5			26.5				42.7		
Mean	1025	72.0			18.1			10.1			5.7			25.9				43.7		
Age group						< 0.01			< 0.01			0.07			< 0.01				< 0.01	
60–69	413	-			19.8	a		10.8	a		5.8	-		26.2	a			44.3	a	
70–79	497	-			18.0	b		10.0	b		5.5	-		25.9	a			43.9	a	
80–89	115	-			12.7	c		8.0	c		6.5	-		25.0	b			41.1	b	
Sex			0.73			< 0.01			< 0.01			< 0.01			0.03				< 0.01	
Male	432	71.9			19.9			10.3			5.2			26.1				46.5		
Female	593	72.0			16.9			10.0			6.1			25.8				41.7		
Diet			0.27			< 0.01			0.11			< 0.01			< 0.01				0.58	
Poor	116	72.5			16.5			9.8			8.1			23.5				43.4		
Good	909	71.9			18.3			10.2			5.4			26.2				43.8		
Sleep						0.97			0.05			< 0.01			< 0.01				0.64	
Poor	280	72.0	0.97		18.1			9.9			7.7			25.4				43.1		
Good	745	72.0			18.1			10.2			5.0			26.1				44.5		
Overweight			0.22			0.62			0.14			0.59			< 0.01				< 0.01	
No	559	72.2			18.2			10.2			5.7			25.1				41.9		
Yes	466	71.8			18.0			10.0			5.8			26.8				44.0		
Education			< 0.01			< 0.01			< 0.01			0.24			< 0.01				< 0.01	
Illiteracy	156	75.7	a		10.6	a		8.8	a		6.2	-		25.4	a			40.1	a	
Primary	241	72.0	b		15.8	b		9.9	b		5.8	-		25.7	ab			43.2	b	
Junior	347	71.0	b		19.8	c		10.3	bc		5.8	-		26.1	bc			44.6	bc	
Senior	222	71.0	b		21.9	d		10.8	cd		5.4	-		25.9	ab			44.7	bc	
college	59	72.0	b		23.6	e		11.1	d		4.7	-		26.6	c			45.8	c	
Living alone			< 0.01			< 0.01			< 0.01			< 0.01			0.06				< 0.01	
No	845	71.5			18.6			10.3			5.4			26.0				44.1		
Yes	180	74.35			15.9			9.4			7.1			25.6				41.9		

MoCA, Montreal Cognitive Assessment; SPPB, Short Physical Performance Battery; GDS, Geriatric Depression Scale; MNA, Mini Nutritional Assessment; BMD, bone mineral density; IQR, interquartile range

*If ANOVA revealed a significant difference, then Student’s Newman-Kuels (SNK) method was used to perform a multiple-comparisons analysis. Different letters (a and b) indicate significant differences between the two groups

The five continuous dependent variables were transformed into dichotomous variables for further sub-health analysis. The percentages of sub-health in women were slightly higher than those in men, but the differences between men and women were nonsignificant (all *P* > 0.05) (Table 2).

**Table 2:** Percentage of sub-health in different sociodemographic groups

	N	MCI (%)	Frailty (%)	Depression (%)	Malnutrition (%)	Osteoporosis (%)
Total		76.2	26.5		15.9	28.6
Age group						
60–69	413	70.5	10.9	11.9	13.6	22.5
70–79	497	78.7	29.8	11.9	15.3	29.0
80–89	115	86.1	68.7	13.9	27.0	48.7
*P*		< 0.01	< 0.01	0.81	< 0.01	< 0.01
Sex						
Male	432	74.3	24.3	11.3	13.4	27.3
Female	593	77.6	28.2	12.7	17.7	29.5
*P*		0.23	0.17	0.53	0.06	0.44
Diet						
Poor	116	85.3	31.9	26.7	51.7	29.3
Good	909	75.0	25.9	10.2	11.3	28.5
*P*		0.01	0.17	< 0.01	< 0.01	0.85
Sleep						
Poor	280	75.0	30.0	23.2	23.9	30.4
Good	745	76.6	25.2	7.9	12.9	27.9
*P*		0.58	0.12	< 0.01	< 0.01	0.44
Overweight						
No	559	75.9	25.0	11.3	24.0	32.0
Yes	466	76.6	28.3	13.1	6.2	24.5
*P*		0.78	0.24	0.14	< 0.01	< 0.01
Education						
Illiteracy	156	69.9	48.1	11.5	23.1	41.0
Primary	241	75.1	32.8	10.0	17.0	29.5
Junior	347	86.7	23.3	13.5	13.0	23.3
Senior	222	69.8	14.0	13.5	16.2	28.8
college	59	59.3	10.2	8.5	8.5	22.0
*P*		< 0.01	< 0.01	0.60	0.03	< 0.01
Living alone						
No	845	75.7	23.4	10.5	15.5	27.0
Yes	180	78.3	41.1	19.4	17.8	36.1
*P*		0.46	< 0.01	< 0.01	0.45	0.01

MCI: Mild cognitive impairment

**Fig. 1:**
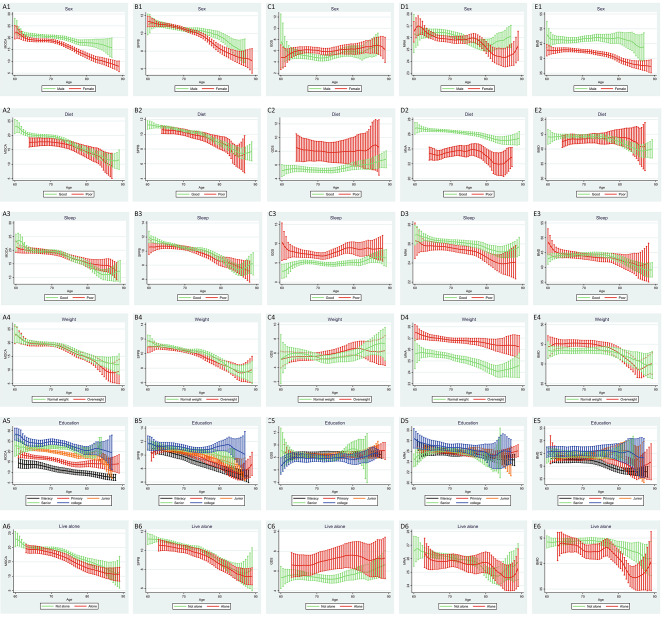
Kernel-weighted local polynomial regression smoothing curves of health assessments and age in different covariates groups **A)** Sub-figures of correlations between MoCA and age, grouping according to sex (A1), diet (A2), sleep (A3), weight (A4), education (A5) or live alone (A6) **B)** Sub-figures of correlations between SPPB and age, grouping according to sex (B1), diet (B2), sleep (B3), weight (B4), education (B5) or live alone (B6) **C)** Sub-figures of correlations between GDS and age, grouping according to sex (C1), diet (C2), sleep (C3), weight (C4), education (C5) or live alone (C6) **D)** Sub-figures of correlations between MNA and age, grouping according to sex (D1), diet (D2), sleep (D3), weight (D4), education (D5) or live alone (D6) **E)** Sub-figures of correlations between BMD and age, grouping according to sex (E1), diet (E2), sleep (E3), weight (E4), education (E5) or live alone (E6)

The kernel-weighted local polynomial smoothing curve clearly demonstrated age-related decreases in the MoCA, SPPB, MNA, and BMD results in different subgroups (sex, diet, sleep, overweight, education, and living alone). The unparallel kernel curves among the subgroups indicated possible interactions. Figure 1A1, 1B1, and 1E1 indicate the possible interactions between age and sex. The GDS scores did not exhibit age-related changes, but the different groups of sex, diet, sleep, and living alone exhibited different levels of scores (Fig. 1).

The results of the multivariate linear regression models were similar to those of the ANOVA and kernel curves. Sociodemographic factors and lifestyle had different effects on the scores of the five assessments. Increased age (*P* < 0.01), female sex (*P* < 0.01), poor dietary habits (*P* < 0.01), lower education level (*P* < 0.01), and living alone (*P* = 0.04) were correlated with lower MoCA scores. Increased age (*P* < 0.01), female sex (*P* < 0.01), lower education level (*P* < 0.01), normal weight (*P* = 0.02), and living alone (*P* = 0.04) were correlated with lower SPPB scores. Female participants with poor dietary and sleep habits and those who were living alone had lower GDS scores (all *P* < 0.01). Female (*P* = 0.01) participants with an increased age (*P* < 0.01) and lower education level (*P* = 0.03) had lower MNA scores. Female (*P* < 0.01) participants with an increased age (*P* < 0.01), normal weight (*P* < 0.01), lower education level (*P* < 0.01), and living alone (*P* = 0.04) had worse BMD results. Sex and age had modified effects on the MoCA score (*P* = 0.03), SPPB score (*P* < 0.01), and BMD (*P* < 0.01). Sex and diet had a modified effect on the GDS score (*P* = 0.03) (Table 3).

**Table 3:** Potential factors related to five health assessments using multiple linear regression models

	MoCA	SPPB	GDS	MNA	BMD
	*P*	*P*	*P*	*P*	*P*
Age (continuous)	< 0.01	< 0.01	0.09	< 0.01	< 0.01
Sex	< 0.01	< 0.01	< 0.01	0.01	< 0.01
Diet	< 0.01	0.27	< 0.01	< 0.01	0.77
Sleep	0.05	0.08	< 0.01	< 0.01	0.21
Overweight	0.25	0.02	0.26	< 0.01	< 0.01
Education	< 0.01	< 0.01	0.36	0.03	< 0.01
Living alone	0.04	0.04	< 0.01	0.94	0.04
Age×Sex	0.03	< 0.01	-	-	< 0.01
Sex×Diet	-	-	0.03	-	-

MoCA, Montreal Cognitive Assessment; SPPB, Short Physical Performance Battery; GDS, Geriatric Depression Scale; MNA, Mini Nutritional Assessment; BMD, bone mineral density

## Discussion

Aging is a complex phenomenon that involves physiological and psychological changes associated with social conditions. This study demonstrated that cognitive and physical function, nutritional status, and BMD decreased with advancing age. Age-specific effects on health states have been supported by other studies [16–20]. In this study, women had worse health status than men based on the assessments of MoCA, SPPB, GDS, MNA, and BMD. Studies on healthy life expectancy in some low- and middle-income countries indicated that women live with more chronic illnesses, depression, or disabilities than men in old age [21, 22]. Although the life expectancy of women is 4.5 years higher than that of men in Shanghai, gains in life expectancy do not necessarily mean better health status. In Shanghai, the mean age at marriage is 2 years lower for women; thus, women are expected to live as widows for 6.5 years. For older women, widowhood is associated with increased vulnerability to loneliness, which may sometimes result in anxiety and depression [23]. Once women depressed more persistently, there would be a lower probability of death [24]. A possible explanation is that low mood may be useful in decreasing motivation and activity when action would be futile or dangerous [25]. The interaction effects between age and sex on the MoCA score, SPPB score, and BMD suggest that more attention should be paid to improve early intervention for elderly women.

Educational level influenced all of the four assessments, except for the GDS. The majority of residents in this suburban district of Shanghai were farmers. Approximately 40% of the participants were illiterate or had only a primary school education. A lower educational level would influence the overall MoCA score because of the difficulty in reading, understanding and calculating the questions in the scale [12, 26]. Educational achievement may be limited by the experience of early-life adversity, which could also influence health later in life, thus confounding associations between education and physical capacity [26, 27]. Studies have highlighted the necessity for cross-cultural considerations on MoCA and suggested appropriate cutoffs and point adjustments for education [12, 28]. With the education-specific cutoff points of the Chinese version of MoCA, the percentage of MCI in junior middle school became the highest among all education groups. Further research should be conducted on the education-specific cutoff points of the MoCA for MCI identification in China. Although the means of GDS increased from 4.7 in the illiteracy group to 6.2 in the college group, the difference did not reach significance (*P* = 0.24). The result was similar to another study conducted in a hospital in Shanghai, China and a study in North India [29, 30]. However, some studies found that educational level had an effect on depression [31, 32]. In our study, the percentage of illiteracy was 42.6% in the 80–89 years age group. When a group is not the minority, illiteracy status might not significantly affect their mentality. Based on the total MNA score, nutritional score did not show an association with BMD. We further analyzed the intake of protein or dairy products in the MNA scale and did not demonstrate that it had any effect on BMD. Self-reported data limited the precision of the information on nutrition, which resulted in nondifferential classification. Furthermore, calcium absorption includes many complicated processes, and nutrition, exercise, and chronic diseases may influence BMD to varying degrees. A systematic review indicated that supplementation with vitamin D alone or with calcium had no significant effect on all-cause mortality [33].

Living alone had negative effects on MoCA, SPPB, and BMD, particularly GDS. Living alone results in less variety in diet, a greater possibility of irregular life, less communication with family members, more social isolation, and more emotional loneliness. Cohort studies and meta-analysis demonstrated that living alone and social isolation were associated with cognitive decline and increased mortality in older adults [34–37]. Recently, a further study on neurobiological mechanisms revealed that social isolation was related to lower gray matter volumes coupled with different molecular functions. These structural differences partly mediated the association between social isolation and an increased risk of dementia, and 75% of the relationship is attributable to depressive symptoms [38]. A study in Korea supported that individuals in rural single-person households had significantly lower BMD and greater odds of osteoporosis in their lumbar spine than urban households with two or more individuals [39]. Elderly individuals living with a spouse or in two-generation households benefited cognitively from internet access [40]. Active cognitive interventions could provide possible benefits to improve cognition, GDS, and functional abilities for community-dwelling elderly living alone [41].

When the five continuous dependent variables were dichotomized based on the sub-health criteria, sex no longer showed the effect. The differences between males and females were small, and categories, particularly dichotomization, lose a lot of continuous variable information [42]. Furthermore, dichotomization leads to underestimation of effect size and loss of measurement reliability [43]. Simple, rapid, sensitive, and specific screening tests for evaluating physical and mental function in geriatric evaluation programs are important for identifying the necessity for early therapeutic intervention. In our study, the MoCA, SPPB, and GDS were applied conveniently and could detect age-related decline or other influencing factors. Both the MoCA and Mini-Mental State Examination (MMSE) were used in this study. Although the MMSE is a commonly used method in cognitive impairment detection as well, the ceiling effect and poor normality in this study were problematic as other studies had pointed out [44, 45]. MoCA was superior to MMSE in the detection of MCI, with higher sensitivity in multiple study settings [46, 47]. Therefore, we did not display the MMSE data. In addition, in our study, the positively skewed distribution of SPPB decreased discriminability. Future research should focus more on optimizing the evaluation of physical function.

## Conclusions

This study analyzed age-related degradation in cognitive and physical function. Both sex and living alone were associated with the MoCA, SPPB, and GDS scores. It is imperative to develop a more sensitive evaluation of physical function, and to encourage various intellectually and emotionally stimulating social activity strategies to promote healthy aging, especially in elderly females and those living alone with a low education level.

## Data Availability

The datasets during and/or analysed during the current study available from the corresponding author on reasonable request.

## Abbreviations

ANOVA: analysis of variance
BMD: bone mineral density
BMI: body mass index
GDS: Geriatric Depression Scale
LPF: lower physical function
MCI: mild cognitive impairment
MMSE: Mini-Mental State Examination
MNA: Mini Nutritional Assessment
MoCA: Montreal Cognitive Assessment
RMN: risk of malnutrition
SPPB: Short Physical Performance Battery
